# Education for public health 2030: transformation to meet health needs in a changing world

**DOI:** 10.3389/fpubh.2023.1269272

**Published:** 2023-12-15

**Authors:** Lisa M. Sullivan, Elizabeth M. Weist, Wendy E. Barrington, Amy L. Fairchild, Wenke Hwang, Marc T. Kiviniemi, Shan D. Mohammed, Victoria A. Wyant, Linda A. Alexander, Laura Magaña

**Affiliations:** ^1^Boston University School of Public Health, Boston, MA, United States; ^2^Association of Schools and Programs of Public Health, Washington, DC, United States; ^3^Center for Anti-Racism and Community Health, Health Systems and Population Health Epidemiology, University of Washington School of Public Health, Seattle, WA, United States; ^4^Maxwell School of Citizenship and Public Affairs, Syracuse University, Syracuse, NY, United States; ^5^Department of Public Health Sciences, Pennsylvania State University College of Medicine, Hershey, PA, United States; ^6^Department of Health, Behavior and Society, University of Kentucky College of Public Health, Lexington, KY, United States; ^7^DEI Educational and Student Initiatives, Northeastern University Bouvé College of Health Sciences, Boston, MA, United States

**Keywords:** academic public health, inclusive excellence, curricular transformation, higher education, pedagogy, academic practice partnerships, graduates

## Abstract

Education for public health is at a critical inflection point, and either transforms for success or fails to remain relevant. In 2020, the Association for Schools and Programs of Public Health launched an initiative, Framing the Future 2030: Education for Public Health (FTF 2030) to develop a resilient educational system for public health that promotes scientific inquiry, connects research, education, and practice, eliminates inequities, incorporates anti-racism principles, creates and sustains diverse and inclusive teaching and learning communities, and optimizes systems and resources to prepare graduates who are clearly recognizable for their population health perspectives, knowledge, skills, attitudes, and practices. Three expert panels: (1) Inclusive excellence through an anti-racism lens; (2) Transformative approaches to teaching and learning; and (3) Expanding the reach, visibility, and impact of the field of academic public health are engaged in ongoing deliberations to generate recommendations to implement the necessary change. The article describes the panels’ work completed thus far, a “Creating an Inclusive Workspace” guide, and work planned, including questions for self-evaluation, deliberation, and reflection toward actions that support academe in developing a resilient education system for public health, whether beginning or advancing through a process of change. The FTF 2030 steering committee asserts its strong commitment to structural and substantial change that strengthens academic public health as an essential component of a complex socio-political system. Lastly, all are called to join the effort as collaboration is essential to co-develop an educational system for public health that ensures health equity for all people, everywhere.

## Introduction

Education for public health is currently at a crucial crossroad, where its members must decide between adapting and thriving, or potentially failing to prepare learners to protect the health of the public. In response to this challenge, the Association of Schools, and Programs of Public Health (ASPPH) launched Framing the Future 2030: Education for Public Health (FTF 2030) in 2020 to advance “equitable, quality education in public health for achieving health equity and well-being for everyone, everywhere.” Conceived prior to COVID-19 and the murder of George Floyd, FTF 2030 is powerfully informed by these events and other realities in seeking a proactive approach to positioning academic public health as a vital contributor in assuring health within a complex and ever-evolving world.

FTF 2030 builds on a prior ASPPH initiative, Framing the Future: The Second Hundred Years of Education for Public Health (2011–2015) (1). Planned in conjunction with the 100-year anniversary of academic public health in the United States, this first effort also sought to review the state of the educational system for public health to better prepare graduates for changes in the global marketplace. It included over 200 members from across the spectrum of public health players who recommended change in seven areas:

- The Master of Public Health Degree: Transitioning to a 21st Century Model.
- The Doctor of Public Health Degree: Preparing Transformative Leaders.
- Undergraduate Education in Public Health: A Dynamic Foundation.
- Incorporating Population Health into Other Professional Degree Programs.
- Community Colleges and Public Health: Providing Pathways to Public Health Education.
- Workforce Development: Bolstering the Governmental Public Health Workforce.
- Blue Ribbon Employers Advisory Board Report: Trends in Public Health Education.

Continually ensuring that education in public health supports the workforce in contributing to a healthier planet necessitates vigilance and constant realignment given changing social, political, technological, and other contexts. In the United States, late 19th and early 20th century pioneers for public health training advocated for hygiene instruction at multiple levels, including practical training in sanitation within medical schools that would confer a diploma of public health (2) and grounding public health in service to communities at four different levels of practice: teacher, research scholar, technical expert, or administrator (3). The subsequent Welch-Rose Report of 1915 defined an “institute of hygiene” that could produce a new cadre of public health professionals and soon after led to creation of the first formal academic institutions of public health in the United States (4).

Education in public health expanded throughout the 20th century while questions about its role, status as a profession, and the requisite content and methods for its teaching and learning resulted in incremental changes but lacked larger structural transformation (4–6). Calls for change in the 1980s and 1990s such as for more comprehensive Doctor of Public Health Training for creating public health leaders (7) and a stronger focus on service learning (8) were followed in the early 2000s by recommendations to ground public health in an ecological framework and new core competencies (9) including climate change (10). Additionally, calls for institutional and instructional reforms that could enable health professions education to better fit health system needs, integrating teaching and learning across disciplines, enhancing lifelong learning to enable acquisition of the enormous body of knowledge for successful practice (11–13), and the increasing interprofessional education (12, 14, 15) stimulated further change.

In the wake of COVID-19, important voices reflected on: education as a collective enterprise (16), the value of combining an eco-social framework with a life course perspective on population health (17), the imperative to combat health inequities (18, 19), and the role of undergraduate education in public health as filling a valuable contribution to the workforce (20). FTF 2030 is building on this rich historical legacy, synthesizing it with our panels’ collective research, observations, and conversations with members and partners in three ASPPH FTF 2030 town halls and other venues to illuminate planning for educational transformation. Of particular note, FTF 2030 is paying special attention to health and social inequities exacerbated by COVID-19, our long overdue racial reckoning, increased gun violence, lack of access to quality healthcare, generational poverty, and catastrophic climate events as important and complex health-related components of our dynamic world and of central relevance to the work of public health professionals.

While interest in public health across the educational continuum increased during COVID-19 (21), so did distrust, even hostility, toward public health practitioners. Despite awe-inspiring scientific advances in fighting the COVID-19 virus, we witnessed a growing mistrust of science and public health practice and are concerned at the forecast that the US stands to lose almost half of its public health workforce by 2025 (22).

In exploring how academic public health is generating learning, readying learners to protect health, and communicating effectively to advance health, the FTF 2030 steering committee (SC) recognizes how public health was challenged in laying out policy choices in the COVID-19 pandemic against a backdrop of core values, priorities, and inevitable tradeoffs that shape decision-making in the face of scientific uncertainty. We saw how our graduates in the workforce were able to contribute and where they lacked the ability to protect the public’s health. We saw how various system breakdowns—from supply chains, to communications, to equitable access to therapeutics, vaccines, and other health protections—harmed population health and raised questions about our professional credibility and cause.

The SC consulted many sources including the 2030 Sustainable Development Goals, which encourages higher education to exert a stronger social presence and engagement in society through purposeful partnerships (23). At a time when trust in higher education is also eroding and political polarization is on the rise, the SC supports universities in reclaiming their position in society as learning spaces for positive change, where critical issues are openly debated and discussed, toward solving complex problems in respectful collaboration with vested partners (24).

## FTF 2030 vision and its expert panels

The SC began their work by formulating a vision, grounded in ASPPH’s strategic plan to deliver equitable, quality education for public health for achieving health equity and well-being for everyone, everywhere (25). ASPPH’s values are explicit and comprised of: diversity, equity, inclusion, and social justice; collaboration; excellence; innovation; agility; and a commitment to public health (25). The SC articulated an aspirational, but achievable, goal: a resilient educational system for public health that promotes scientific inquiry, connects research, education, and practice, eliminates inequities, incorporates anti-racism, creates and sustains diverse and inclusive teaching and learning communities, and optimizes systems and resources to prepare graduates who are clearly recognizable for their population health perspectives, knowledge, skills, attitudes, and practices. The SC also outlined strategies and principles to realize the vision (see Supplementary Table 1).

The SC is engaging in critical examinations of the multiplicity of factors that transform education by centering on the roles of five key drivers—university systems and structures; faculty; staff; students; and partners—in improving structures and systems to better enable academic public health to serve health and wellness. Furthermore, FTF 2030 is drawing on recent work of fellow ASPPH task forces: the Scholarship of Teaching and Learning (26), Zero Tolerance of Harassment and Discrimination (27), Dismantling Racism and Structural Racism in Academic Public Health (28), Responding to the Climate Change Crisis (29), and Gun Violence Prevention (30) to iterate and synchronize their collective work.

To develop tactics, measures, and outcomes for each of the strategies and principles, the SC identified three cross-cutting areas to focus efforts:

1. Inclusive excellence through an anti-racism lens;
2. Transformative educational models and pedagogy; and
3. Expanding the reach, visibility, and impact of the field of academic public health.

In 2021, the SC populated and charged three cross-cutting expert panels to address each focus area. Each panel is charged with assessing the current state of the environment of education for public health, envisioning future developments encompassing inherent uncertainty and the complexity of systems and structures, and developing promising practices and exemplars addressing these strategies along with principles for schools and programs to model or adapt, as appropriate. The three expert panels are communicating broadly as part of a cohesive approach including annual town halls, sessions with partners, and a recent Teaching Public Health Conversation (31).

**Figure 1:**
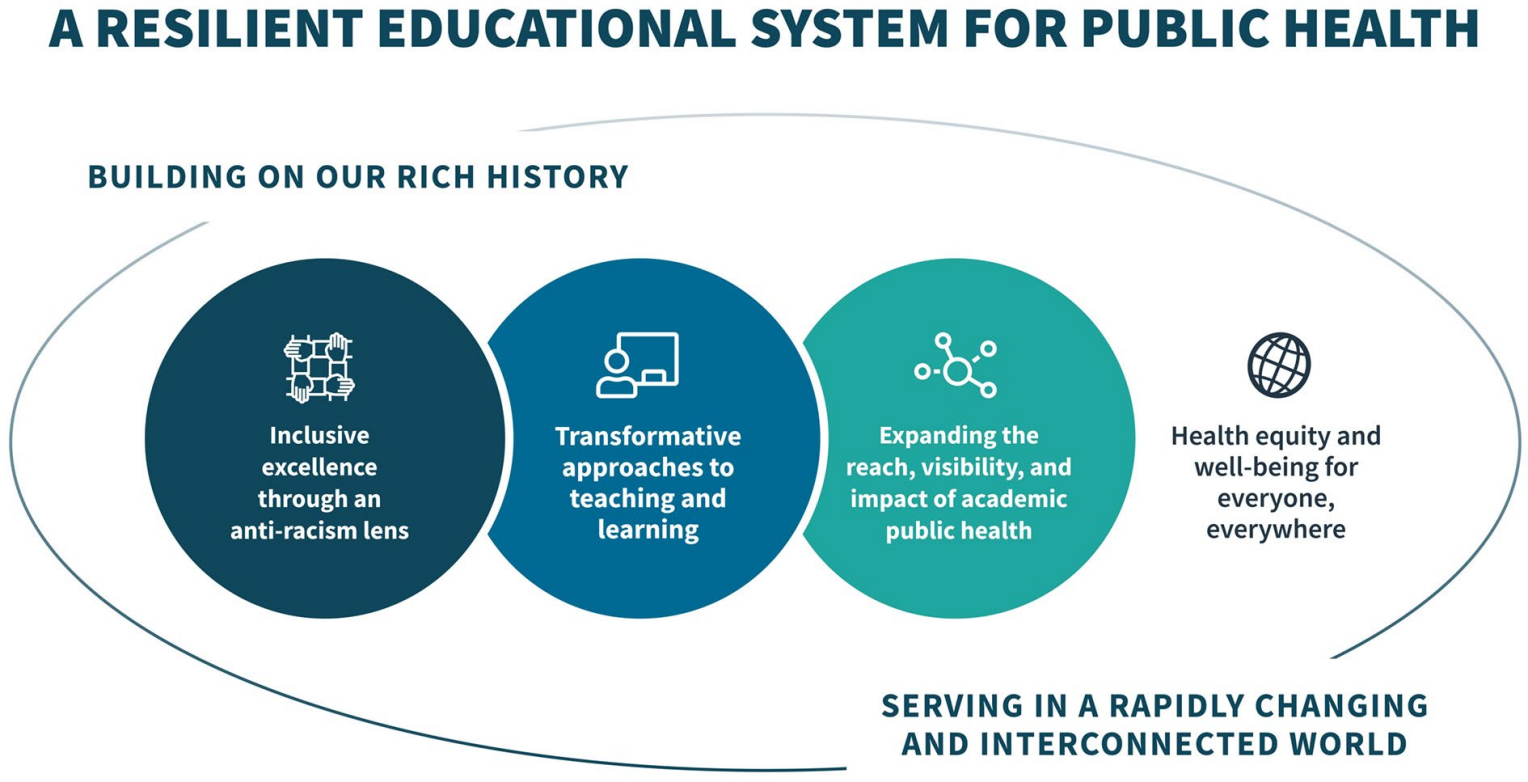
Framing the future 2030 cross-cutting expert panels,

The panels view inclusive excellence through an anti-racism lens as foundational and essential to the success of this effort. Without inclusive representation, perspectives, and experiences from all people, we cannot envision transformative educational models and pedagogy that are necessary to meet the needs of the field now and into the future. Likewise, without inclusive representation and engagement in more effective educational models and pedagogy, we cannot possibly expand the reach, visibility, and impact of academic public health (see Figure 1).

## FTF 2030: Inclusive excellence through an anti-racism lens

The Inclusive Excellence through an Anti-racism Lens panel includes 14 representatives from academe and practice, who in their formation process developed a “Creating an Inclusive Workspace” guide their work (32). This document articulates the panel’s values, norms, and principles to direct their efforts, and includes their commitment to a participatory decision-making process. With the understanding that the process would adjust as the group matured, the panel offers their process as an example for potential adaptation or adoption by groups advancing similar efforts.

The panel worked together to define key terms including developing a collective understanding of what is meant by inclusive excellence through an anti-racism lens in higher education. The definitions have been expanded and attached to examples in a comprehensive glossary that builds on other work and will soon be shared.

After completing these foundational steps, the panel undertook an environmental scan of promising practices, underway or planned, to promote inclusive excellence across each of the five drivers. The purpose is to identify what success looks like in a sample of ASPPH-member institutions for surfacing working models and lessons learned. Concrete, living examples, along with the institutions where they were implemented, will be posted for others to review, adopt or adapt, and connect on mutual efforts. The intent is to build a resource for the community, and to invite others to contribute their own models or adaptations to add to a dynamic repository for the benefit of all.

## FTF 2030: Transformative approaches to teaching and learning

The Transformative Approaches to Teaching and Learning expert panel includes 17 representatives from academe and practice who began their work by identifying four workstreams: Foundational skills and values needed by public health professionals to succeed in the current socio-political context; Teaching (pedagogy) and learning; Interacting with those outside of academic public health; and Content delivery and pathways. The workstreams’ discussions paved the way for a forthcoming framing document with transformative approaches to teaching and learning in public health, the associated assessment of learning outcomes, and recommendations to deliver on the change.

The panel calls for a paradigm shift that requires more than teaching new knowledge and skills. It requires creating robust learning spaces with new ways of teaching, new opportunities tailored to learners across the life-course, and preparing graduates with not only science-based content but with a strong grounding in public health values and nuanced skills to engage in authentic deliberation and advocacy across sectors, professions, and communities for policies, programs, practices, and services that protect the people’s health. As we in public health recognize the need for more upstream health interventions, the panel is addressing the need to prepare students to grapple with the root causes of health in complex systems to enable them to shape public policy conversations about population health. And just as the recommendations of the original Framing the Future initiative featured prominently in the Council on Education for Public Health criteria, the FTF 2030 leaders plan again to work collaboratively in advising on criteria changes to improve learner readiness to address public health challenges, with a renewed focus on values, ethics, and advocacy skills to contribute effectively to social justice and health equity.

## FTF 2030: Expanding the reach, visibility, and impact of academic public health

The Expanding the Reach, Visibility, and Impact of Academic Public Health expert panel includes 13 representatives from academe, private industry, public health practice, and a philanthropic organization who began their work together by developing a series of guiding questions to shape recommendations for change. A core principle for recommending change is that academic public health should approach community engagement from a perspective of humility versus the “we are the experts” approach that has too often driven past efforts. The panel is stressing the urgency to rebuild trusting relationships with the community and to prepare for the next public health emergency as core goals for action. These aims point to the need for transformation in two areas: first, a deliberate focus on what communities and partners need from us in academic public health, and not the opposite, including, but not limited to, how we engage younger learners in public health and how to ensure public health is “at the table” in interprofessional educational for collaborative practice; and, second, education and training, curricular change, and competencies that better prepares graduates and that meets professionals where they are in terms of their continuing education needs while drawing from their expertise and assets in such training for protecting health. The panel’s recommendations will be shared in a white paper for comment, as will examples of promising practices and recommendations for short- and long-term action.

## Deliberative reflection

A critical issue, relevant to all three expert panels, is ensuring that FTF 2030 products are accessible and useful to all member schools and programs served by ASPPH. Understanding that members operate in unique contexts and with differing core missions, financial models, and other organizational factors, flexibility needs to be baked into recommendations. In addition to the work products outlined above, the SC is drafting questions for self-evaluation, deliberation, and reflection toward actions that support member schools and programs in approaching these issues and beginning or advancing through a process of change. The questions are aimed to support a participatory visioning and planning process whereby interested members could find answers for themselves that best fit their situations in responding to FTF 2030 recommendations.

The questions will “invite consideration for what it means to be responsive to what’s happening in the world and how it makes its way into our classroom or our community learning spaces and, at a deeper level…how our choices influence effectiveness and sustainability (16).” It will include specific questions for the key drivers, honing in on the strategies and principles outlined in Supplementary Table 1. This approach is intended to support members and guide them in drawing upon their own lived experiences and data in planning for implementation and change. The questions are intended to generate creative and meaningful conversations and self-reflection, with the ultimate goal of identifying, very specifically, what success means and what it could look like in each specific culture and context. Similar to all other products of this effort, the draft questions will be shared for feedback and modification.

## Discussion

The FTF 2030 effort is an opportunity to create a future educational system for public health that is inclusive, equitable, innovative, adaptive, and sustainable. The work required to do this cannot remain static nor can it occur in isolation. Academic public health will not deliver in protecting public health if constituents work within their schools and programs alone, in familiar, comfortable spaces. To transform education to meet health needs not only for today, but for tomorrow, schools and programs must work together and strengthen existing outreach and create new partnerships if we are to be successful.

We are deep into a period of great disruption and have many options for transformation. During the COVID-19 pandemic, we had to change and adapt in ways and at a cadence that we never imagined possible. We learned much about what worked and what did not work to promote student learning, methods to use educational technology more effectively, and the importance of remaining true to our core values. We also observed greater injustices, widening gaps, in education and in most other sectors. While we acknowledge that this is a moment of profound social and political uncertainty and with existential challenges to core public health principles along with new barriers to making progress on health and social equity, we remain undaunted in our commitment to work for change. We are redirecting our energies toward structural and substantial change that strengthens academic public health as an essential component of a complex socio-political system that ensures health and well-being for everyone, everywhere.

## Call to action

With support from ASPPH, the SC is committed to amplifying existing, positive efforts by the ASPPH-member schools and programs and improving this work across sectors, professions, and politics. Products of the FTF 2030 initiative are disseminating in real-time and all partners are not only welcomed but needed to join this effort by contributing feedback at academics@aspph.org to improve our work and to make it more useful for all. A key question we continue to ask ourselves and post to readers is: Are we bold enough?

We invite all interested in advancing promising strategies and initiatives for creating a more just and healthier world to collaborate in sharing lessons learned and developing metrics to monitor shared progress. Everyone is necessary and essential for building a common evidence-base to inform future decision-making.

As our core principles of diversity, inclusivity, equity, innovation, resilience, and social justice continue to be challenged, we must respond to the scrutiny through organized, collaborative efforts. We need more open conversations to surface all perspectives, and to ensure that strategies to advance the health of all are based on science and evidence and that we co-communicate our messages effectively and respectfully. We need to strengthen education for public health, collaborating with all players for enabling health decisions to draw from relevant and timely data so individuals and communities can respond effectively. This is how we could contribute to a culture of trust and improved health and wellness. And, at this important socio-political inflection point, those of us who can do more, must do more to create a more just and healthier world.

## Data availability statement

The original contributions presented in the study are included in the article/supplementary material, further inquiries can be directed to the corresponding author.

## Author contributions

LS: Conceptualization, Methodology, Writing – original draft, Writing – review & editing. EW: Conceptualization, Methodology, Project administration, Writing – original draft, Writing – review & editing. WB: Conceptualization, Methodology, Writing – review & editing. AF: Conceptualization, Methodology, Writing – review & editing. WH: Conceptualization, Methodology, Writing – review & editing. MK: Conceptualization, Methodology, Writing – review & editing. SM: Conceptualization, Methodology, Writing – review & editing. VW: Conceptualization, Methodology, Project administration, Writing – review & editing. LA: Conceptualization, Methodology, Writing – review & editing. LM: Conceptualization, Methodology, Writing – review & editing.

## References

[1] Petersen DJ Weist EM. Framing the future by mastering the new public health. J Public Health Manag Pract. (2014) 20:371–4. doi: 10.1097/PHH.0000000000000106 24813674 PMC4032212

[2] Sedgwick WT. Report of committee on the teaching of hygiene and granting a diploma of doctor of public health. Public Health Pap Rep. (1902) 28:170. 19601048 PMC2329443

[3] Rosenau MJ. Courses and degrees in public health work. J Am Med Assoc. (1915) LXIV:794–6. doi: 10.1001/jama.1915.02570360010003 25781455

[4] Welch W. The welch-rose report: a public health classic, a publication by the delta omega alpha chapter to mark the 75th anniversary of the founding of the Johns Hopkins University School of hygiene and public health, 1916–1992. Washington: Delta Omega Honorary Public Health Society (1992).

[5] Evashwick CJ Begun JW Finnegan JR. Public health as a distinct profession. J Public Health Manag Pract. (2013) 19:412–9. doi: 10.1097/PHH.0b013e31828002d2 23896977

[6] Fee E Acheson RM. A history of education in public health: health that mocks the doctors’ rules. A history of education in public health: Health that mocks the doctors’ rules. Oxford: Oxford University Press (1991).

[7] Roemer MI. Preparing public health leaders for the 1990s. Public Health Rep. (1988) 103:443. 3140268 PMC1478128

[8] O'Neil EH. Health professions education for the future: Schools in service to the nation. San Francisco: Pew Health Professions Commission (1993) 2.

[9] Hernandez LM Rosenstock L Gebbie K. Who will keep the public healthy?: educating public health professionals for the 21st century. (2003). 25057636

[10] Evans D. The role of schools of public health: learning from history, looking to the future. J Public Health. (2009) 31:446–50. doi: 10.1093/pubmed/fdp065, PMID: 19574273

[11] Frenk J Chen L Bhutta ZA Cohen J Crisp N Evans T. Health professionals for a new century: transforming education to strengthen health systems in an interdependent world. Lancet. (2010) 376:1923–58. doi: 10.1016/S0140-6736(10)61854-5 21112623

[12] Rosenstock L Helsing K Rimer BK. Public health education in the United States: then and now. Public Health Rev. (2011) 33:39–65. doi: 10.1007/BF03391620, PMID: 32226193 PMC7099377

[13] Sullivan L Galea S. A vision for graduate public health education. J Public Health Manag Pract. (2017) 23:553–5. doi: 10.1097/PHH.0000000000000664 28957902

[14] Goldman L, Editor responding to the climate change and health crisis: a framework for academic public health. APHA 2022 annual meeting and expo, Boston. (2022).

[15] Thibault GE. The future of health professions education: emerging trends in the United States. FASEB Bio Adv. (2020) 2:685–94. doi: 10.1096/fba.2020-00061, PMID: 33336156 PMC7734422

[16] Sullivan LM Velez AA Galea S. Graduate public health education in the post-COVID-19 era. Lancet Public Health. (2020) 5:e473. doi: 10.1016/S2468-2667(20)30181-X 32888441 PMC7462636

[17] Shultz JM Sullivan LM Galea S. Public health: an introduction to the science and practice of population health. New York, NY: Springer Publishing Company (2021).

[18] Frenk J Chen LC Chandran L Groff EO King R Meleis A. Challenges and opportunities for educating health professionals after the COVID-19 pandemic. Lancet. (2022) 400:1539–56. doi: 10.1016/S0140-6736(22)02092-X 36522209 PMC9612849

[19] Magaña L Biberman D. Training the next generation of public health professionals. American Journal of Public Health: American Public Health Association (2022). 579–581. 10.2105/AJPH.2022.306756 PMC8961832 35319952

[20] Riegelman RK. Two decades of progress in undergraduate public health: where do we go from here? American Journal of Public Health: American Public Health Association (2023). 9–11. 10.2105/AJPH.2022.307145 PMC9755945 36516381

[21] Warnick A. Interest in public health degrees jumps in wake of pandemic: applications rise. Nation’s Health. (2021) 51:1–12.

[22] Leider JP Castrucci BC Robins M Hare Bork R Fraser MR Savoia E. The exodus of state and local public health employees: separations started before and continued throughout COVID-19: study examines state and local public health agency employees intent to leave or retire in 2017 with actual separations through 2021. Health Aff. (2023) 42:338–48. doi: 10.1377/hlthaff.2022.01251, PMID: 36877909

[23] Parr A. Knowledge-driven actions: transforming higher education for global sustainability. Paris, France: UNESCO223 (2022).

[24] Spector P Shreve G Daniels RJ. What universities owe democracy. (2021).

[25] Association of Schools and Programs of Public Health. Strategic plan 2030. (2022). Available at: https://aspph-prod-web-assets.s3.amazonaws.com/StrategicFramework2030.pdf.

[26] Association of Schools and Programs of Public Health. Education – scholarship of teaching and learning (SoTL) task force. (2020–2022). Available at: https://aspph.org/education-practice-research/education/.

[27] Halkitis PN Alexander L Cipriani K Finnegan J Jr Giles W Lassiter T. A statement of commitment to zero tolerance of harassment and discrimination in schools and programs of public health. Public Health Rep. (2020) 135:534–8. doi: 10.1177/0033354920921816, PMID: 32353244 PMC7383757

[28] Association of Schools and Programs of Public Health. Dismantling racism and structural racism in academic public health: a framework. Washington, D.C: Association of Schools and Programs of Public Health (2022).

[29] Association of Schools and Programs of Public Health. Responding to the climate change and health crisis: a framework for academic public health Association of Schools and Programs of Public Health 2022 October 2.(2022).

[30] Association of Schools and Programs of Public Health. Gun violence prevention: an academic public health framework. Washington, D.C: Association of Schools and Programs of Public Health (2023).

[31] Boston University. Teaching public health equity for all. (2023). Available at: https://www.bu.edu/sph/conversations/education/teaching-public-health-3/#phc-section-videos

[32] Association of Schools and Programs of Public Health. Creating an inclusive workspace thorugh an anti-racism lens, an example of consensus process in academic public health (2022).

