# Supplementary material for: Education for public health 2030: transformation to meet health needs in a changing world
Source: Front Public Health. 2023 Dec 15;11:1269272. doi: 10.3389/fpubh.2023.1269272 (PMC10757328; doi:10.3389/fpubh.2023.1269272)

Supplementary Material

# Supplementary Figures and Tables

**Table 1. Framing the Future 2030: A resilient educational system for public health**

*University systems and structures* (including school and program governance, leadership, and administration) that promote high-quality, inclusive, and equitable teaching, learning, and practice in public health, including:

- appointment, tenure, and promotion systems and policies that promote inclusive excellence and recognize high-quality education and teaching practices
- institutional support for high-quality: education; learning environments; student recruitment, retention, and support services; and student learning outcomes
- valuing faculty and staff as equal partners in achieving objectives and goals
- infusing public health content in general education curricula and creating pathways to education in public health
- developing and sustaining a culture of social justice, inclusive excellence, and anti-racism

*Faculty* who:

- use advances in the science of teaching and learning throughout the educational process
- contribute to student attainment of competence, professionalism, and confidence, including preparing all students to become effective agents of change
- train the next generation of educators in public health
- promote student learning in various environments
- are trained in and deliver on the commitment to social justice, inclusive excellence, and anti-racism in their teaching, research, practice, and service
- advocate for engaged and evidence-based public health research, translation, dissemination, and practice in the community
- are committed to their own continuous learning and professional development

​​     ​

*Staff* (in administrative roles) who:

- ​​​use high-impact practices in fulfilling their roles and are committed to continuous quality improvement
- are well prepared to recruit, advise, support, and guide diverse learners
- use advances in the science of teaching and learning throughout the educational process
- ​​​​​​​​​​​participate as appropriate in the research, education, and service work of the school/program
- are trained in and deliver on the commitment to social justice, inclusive excellence, and anti-racism in their professional activities
- are committed to their own continuous learning and professional development

*Students, including post-doctoral trainees, and other learners* who:

- appreciate the historical context and organization of public health
- value and can engage in ethics-based, evidence-based public health research and practice
- become resilient, critical, compassionate, constructive, and collaborative advocates, systems thinkers, and problem-solvers
- ​​​​​​​​​​​engage actively with others from diverse backgrounds, settings, professions, and sectors
- are trained in and deliver on the commitment to social justice, inclusive excellence, and anti-racism in their professional activities
- thrive in various learning environments
- demonstrate competencies in advocating for and advancing health and well-being for everyone, everywhere~~.~~

*Partners (in collaboration with academe) who:*

- educate academia on workforce assets and needs by collaborating in preparing public health graduates to deliver on pressing community health issues
- report respectful engagement and who feel heard by their academic colleagues
- co-respect the time it takes and processes required to build relationships
- contribute to recognizing history, context, and actions that perpetuate and disrupt inequities^16^
- share in co-creating values, norms, and principles that govern healthy and productive group interactions^17^
- collaborate bidirectionally in building successful connections to promote and advance population health
- interact as equal partners in design, implementation, and evaluation of interprofessional educational and training activities in contributing to health equity
- amplify the most effective strategies to communicate the value and essential functions of public health to the public, domestically, and globally

## Supplementary Figures

**Supplementary Figure 1.** **Integrating the work of the three FTF2030 expert panels**

##
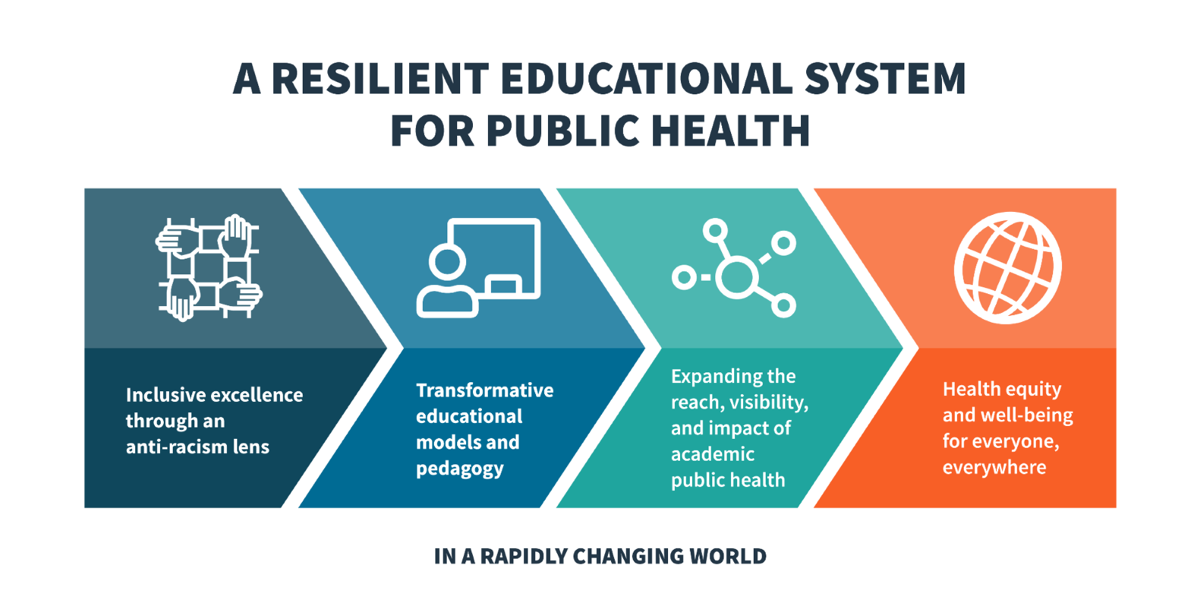

Supplement: Supplementary file 1 [file Data_Sheet_1.docx]
